# Comparative transcriptomics indicates endogenous differences in detoxification capacity after formic acid treatment between honey bees and varroa mites

**DOI:** 10.1038/s41598-020-79057-9

**Published:** 2020-12-14

**Authors:** Antonia Genath, Soroush Sharbati, Benjamin Buer, Ralf Nauen, Ralf Einspanier

**Affiliations:** 1 Institute of Veterinary Biochemistry, Freie Universität Berlin, Berlin, Germany; 2 Bayer AG, Crop Science Division, Pest Control, Monheim, Germany

**Keywords:** Entomology, Transcriptomics

## Abstract

Formic acid (FA) has been used for decades to control *Varroa destructor*, one of the most important parasites of the western honey bee, *Apis mellifera*. The rather unselective molecular mode of action of FA and its possible effects on honeybees have long been a concern of beekeepers, as it has undesirable side effects that affect the health of bee colonies. This study focuses on short-term transcriptomic changes as analysed by RNAseq in both larval and adult honey bees and in mites after FA treatment under applied conditions. Our study aims to identify those genes in honey bees and varroa mites differentially expressed upon a typical FA hive exposure scenario. Five detoxification-related genes were identified with significantly enhanced and one gene with significantly decreased expression under FA exposure. Regulated genes in our test setting included members of various cytochrome P450 subfamilies, a flavin-dependent monooxygenase and a cytosolic 10-formyltetrahydrofolate dehydrogenase (FDH), known to be involved in formate metabolism in mammals. We were able to detect differences in the regulation of detoxification-associated genes between mites and honey bees as well as between the two different developmental stages of the honey bee. Additionally, we detected repressed regulation of *Varroa* genes involved in cellular respiration, suggesting mitochondrial dysfunction and supporting the current view on the mode of action of FA—inhibition of oxidative phosphorylation. This study shows distinct cellular effects induced by FA on the global transcriptome of both host and parasite in comparison. Our expression data might help to identify possible differences in the affected metabolic pathways and thus make a first contribution to elucidate the mode of detoxification of FA.

## Introduction

The ectoparasitic mite *Varroa destructor*, hereafter referred to as *Varroa*, is currently considered the greatest threat of the western honey bee, *Apis mellifera,* and contributes to global colony losses^1,2^. Female mites feed on the fat body tissue of both adult and immature bees^3^, but reproduce only inside sealed worker and drone brood cells^4^.

The mite causes considerable damage to its host, directly by feeding from the fat body tissue^3^ and indirectly by transmitting several viruses^5–8^ and bacteria^9,10^. Infestation leads to weight loss, a shortened lifespan, malformations and weakening of the host^11–14^. If left untreated, a mite-infested colony collapses within 1–3 years^15^.

Since the transfer of *Varroa* from the eastern honey bee, *Apis cerana*, to the western honey bee in the beginning of the nineteenth century, several control strategies have been developed to fight the mite in order to prevent colony losses. Formic acid (FA) is widely used as an alternative treatment throughout the world since it is the only treatment known to affect both phoretic and reproductive mites in sealed brood cells^16^. Further advantages compared with synthetic varroacides are the low risk of developing genetic pest resistance and leaving residues in hive products^17–19^. However, the treatment efficiency varies widely depending on ambient temperature and humidity, colony strength, presence of brood as well as type and position of the evaporator in the hive^1,20,21^. Furthermore, the ‘‘therapeutic index”, the range between the lethal doses for the mites and honey bees is very narrow, resulting in unintended increased queen mortality and negative effects on brood and newly emerged workers^1,22,23^.

Although a considerable number of studies have been conducted regarding the effectiveness under different beekeeping and climatic conditions and the impact on honey bees^20–22,24,25^, surprisingly little is known about the detailed molecular mode of action of FA and the cellular response in honey bees and mites. It is generally assumed that FA exerts its damaging effect by inhibiting the mitochondrial electron transport chain through binding to cytochrome c oxidase^26–28^ and subsequently leads to inhibition of respiration along with hyperacidity of the body^22^. Whereas studies on gene expression of *Varroa* exposed to FA are lacking, one study in honey bees treated with FA (Mite Away) indicates that FA affects the expression of the detoxification related *PKA-C1* gene^29^. Furthermore, FA was found to induce down-regulation of cytochrome P450 (*CYP9Q3*) and up-regulation of *defensin-1*, suggesting a partial impairment of detoxification mechanisms and induction of immune responses of the exposed bees^30^.

This study aims to deepen our understanding of sublethal effects and the underlying molecular detoxification response of *A. mellifera* and *V. destructor* upon FA treatment. We have chosen RNA-Seq to study the transcriptome of this major managed pollinator and one of its most relevant parasites to identify potential target structures and regulated metabolic pathways under FA treatment. This untargeted approach may allow to shed light on unintended and yet unknown cellular effects in the honey bee as well as varroa mites.

## Materials and methods

### Fumigation experiments and sampling

All experiments were conducted in seasons 2018 and 2019 at the Department of Veterinary Medicine at Freie Universität Berlin, Germany (Lat: 52.516181, Long: 13.376935). Four *Apis mellifera carnica* honey bee colonies housed in two storied Segeberger Classic beehives and naturally infested by *Varroa* were used for this study. The colonies had a natural mite infestation. Besides this, they were free of disease and the absence of American foulbrood (*Paenibacillus larvae*) was confirmed by an authorized health certificate. To obtain individuals of the same age, the queen was caged on an empty brood comb 21 and 10 days before the trial to receive either freshly hatched workers (from day 21 after caging; hereafter referred to as workers) or newly capped brood (from day 10 after caging; hereafter referred to as larvae), respectively. Colonies were treated once with 200 ml 60% FA ad us. vet. (Serumwerk Bernburg AG, Bernburg, Germany) by means of a Nassenheider Verdunster universal R (Joachim Weiland Werkzeugbau GmbH & Co. KG, Hoppegarten, Germany). After 12 days from the beginning of the administration, when the entire FA had evaporated, the Nassenheider devices were removed. In 2018, the average daytime temperature was 21 °C and precipitation was 12.2 L per square metre during the treatment period. In the 2019 season, the average daily temperature during the treatment period was 20.3 °C and the average precipitation 28.2 L per square metre.

From each of the four colonies, three randomly chosen workers (can be easily distinguished from older stages by their appearance and behaviour^31^) and larvae from the prepared brood combs were sampled directly before the beginning of FA treatment as untreated control (0 h) and 24 h post treatment period as FA treated group (24 hpt). Subsequently, individuals were stored at − 80 °C until further processing.

The samples analysed in the RNA-Seq were collected during the 2018 season only. In total we collected three biological replicates per colony and treatment group (a total of 24 workers and 24 larvae). RT-qPCR to validate the RNA-Seq data was complemented with the same samples used in RNA-Seq, and also with three biological replicates per colony and treatment group from the 2018 season and three biological replicates per colony and treatment group from the 2019 season (a total of 72 workers and 72 larvae). By collecting several biological replicates per colony, genetic differences within the individual colonies (due to the mating of the queen with several drones, workers are half-sisters^32,33^) and between the colonies should be equalised.

By collecting in different seasons, differences between years, such as weather conditions or different supply of pollen and nectar, should be compensated.

Adult female varroa mites collected across the honey bee colonies mentioned previously were sampled from brood and adult bees at the same time intervals as described above. Mites from capped brood cells were sampled by opening the cell caps and removing individual mites using paintbrushes and tweezers. Additionally, phoretic mites were collected directly from the bodies of adult honey bees and the sticky board surface, which is usually placed underneath the hives to assess natural mite fall. It was ensured that the mites from the sticky boards were still alive. Mites collected from the cells and from the phoretic stage across all four colonies were pooled in groups of ten, placed in 1.5 ml microcentrifuge tubes and stored at − 80 °C until RNA isolation. It should be emphasised that these collection methods provide mites of unknown age that have reproduced in unknown numbers. However, care was taken to ensure that only large (about 1.5 mm wide) and reddish-brown mites were used for further analysis, so as to guarantee the adult stage of the mites^34^. The advantage of this method is that the mites are not affected by treatment with water or icing sugar^34^.

Similar to the honey bee, the samples analysed in the RNA Seq were only collected during the 2018 season. In total, we collected three biological replicates per treatment group (a total of six pools of ten mites collected from all colonies per biological replicate). The RT-qPCR to validate the RNA-Seq data was performed with the same samples used in the RNA-Seq and supplemented with three biological replicates per treatment group from the 2018 and 2019 seasons (a total of nine untreated control groups (0 h) and 9 FA treated groups [24 hpt)]. As before, one biological replicate again consisted of 10 mites at the reproductive and phoretic stage, collected across the four colonies.

### RNA isolation

#### Honey bees

For the RNA extraction we modified the protocol according to Kablau et al.^35^ and chose a slightly different approach for both age groups of the honey bee.

Total RNA from newly emerged honey bees was isolated using an automated homogenizer (BeadBlaster, Benchmark Scientific, Edison, USA) and 1.4 mm ceramic lysing matrix beads (MP Biomedicals, Heidelberg; 3 × 20 s at 7 M; 3 min on ice). The Quick-RNA Microprep Kit (Zymo Research Europe GmbH, Freiburg, Germany) was used, according to the manufacturer's protocol, slightly adjusted by adding an additional centrifugation step for further drying of the matrix.

Each individual larva was homogenised in 1 ml of Trizol with a BeadBlaster (Benchmark Scientific, Edison, USA) and 1.4 mm ceramic lysing matrix beads (MP Biomedicals, Heidelberg) (3 × 20 s at 7 M; 3 min on ice in). After incubation for 5 min at room temperature, samples were centrifuged at 12,000×*g* for 10 min at 4 °C. Five hundred µl of the supernatant was then transferred into an RNase-free tube, while the resulting fat monolayer was carefully avoided. Four hundred μl of chloroform was added, samples vortexed for 15 s, left for 3 min at room temperature, and centrifuged at 12.000×*g* for 30 min at 4 °C. The upper phase was gently transferred into a new tube. Total RNA was extracted following the Quick-RNA Microprep Kit (Zymo Research Europe GmbH, Freiburg, Germany) instructions.

#### Mites

RNA was extracted from pools of ten individual mites sampled across all colonies using the Quick-RNA Microprep Kit (Zymo Research Europe GmbH, Freiburg, Germany) following the manufacturer’s instructions after mechanical homogenization with a micro pestle directly in 300 µl lysis buffer.

RNA quantity and integrity of all samples were determined using the DeNovix DS-11 Spectrophotometer (Biozym Scientific GmbH, Hessisch Oldendorf, Germany) and the Agilent Technologies 2100 Bioanalyser with the RNA 6000 Nano Kits (Agilent Technologies, Waldbronn, Germany). The concentration was then adjusted to 1 μg/μl with RNase-free water and samples stored at − 80 °C until further processing.

### RNA-sequencing experiments

Total RNA was treated with DNase I endonuclease (Thermo Scientific, Karlsruhe, Germany) in a 96 well thermal cycler (Veriti, Applied Biosystems Deutschland GmbH, Darmstadt, Germany). DNase treated RNA (1500 ng) of each sample (in total 54 samples) were submitted to GENEWIZ Germany GmbH, Leipzig, Germany, for library preparation using NEBNext Ultra II DNA Library Prep Kit for Illumina (New England Biolabs GmbH, Frankfurt am Main, Germany) followed by sequencing on an Illumina HISeq4000 System (Illumina Inc., San Diego, United States) following the manufacturer’s protocol. RNA integrity was confirmed by Agilent 4200 Tapestation (Agilent Technologies Inc., Santa Clara, United States) and concentration was assessed by Qubit assay (Thermo Scientific, Karlsruhe, Germany) prior to library preparation. Raw de-multiplexed sequence files were obtained in FASTQ format.

### Analyses of differentially expressed genes

Sequence quality of the RNA-Seq libraries was assessed using FastQC (http://www.bioinformatics.babraham.ac.uk/projects/fastqc/).

The reads were aligned to the latest version of the honey bee reference genome (GenBank: Amel_HAv3.1) and mite reference genome (GenBank: Vdes_3.0), respectively, using STAR aligner v2.6.1d^36^. The quantification of alignments was done using RSEM v1.3.1^37^ and kallisto v0.45.0^38^ resulting in highly correlating results. For downstream analysis, kallisto quantification was used. The Bioconductor DEseq2 package v1.16.1^39^ in the R v3.4.1 environment was used to identify differentially expressed transcript genes in treated versus control group. For larvae, pairwise DEGs were identified for each hive individually and intersected. Due to drop outs of samples of worker bees with high infection rates, a multifactorial model was used to identify DEG after treatment. A principal component analysis (PCA) was performed to clarify general distribution patterns and separations of honey bee and varroa mite gene expression profiles of the different treatment groups by reducing the dimensions of the variables. For functional annotation of the official gene set, Gene Ontology (GO) terms were assigned to individual protein sequence using Blast2GO v1.3.3^40^. Therefore, domains were predicted using InterproScan v5.17-56.0^41^ and genes were searched against Uniprot KB using NCBI-BlastP v2.2.27^42^. GO term enrichment analysis was performed on induced and repressed genes using the Bioconductor package goseq v1.28.0^43^.

RNAseq mass data have been submitted to the NIH resource via SRA submission under the ID SUB7762334.

### RT-qPCR analysis

For validation of RNA-Seq data, RT-qPCR was performed with seven selected genes for honey bees and two selected genes for *Varroa*, which are associated with detoxification and showed a regulated expression in response.

Gene expression experiments were performed as described earlier with few modifications^44^. One μg RNA was treated with DNase I endonuclease (Thermo Scientific, Karlsruhe, Germany) and subsequently reverse-transcribed with RevertAid M-MuLV Reverse Transcriptase (Thermo Scientific, Karlsruhe, Germany) in 20 µl total volume using random hexamers in a 96 well thermal cycler (Veriti, Applied Biosystems Germany GmbH, Darmstadt, Gemany).

Sets of specific primers were designed using the NCBI primer design tool (https://www.ncbi.nlm.nih.gov/tools/primer-blast/) and commercially synthesized (Sigma-Aldrich Chemie GmbH, München, Germany). All primers were designed to cross an exon–exon boundary. The amplification efficiency of each primer pair (Table 1) was assessed by the use of serially diluted PCR products for standard curves and showed suitable efficiency of 90–110% at 60 °C annealing temperature across all targets. Prior to use for RT-qPCR, all primers were confirmed by electrophoresis showing a single expected size band. Each PCR product was then verified by DNA-sequencing (Eurofins GATC, Köln, Germany). Selected candidate genes with corresponding sequences are listed in Table 1.

**Table 1:** Sequences of qPCR primers used in this study.

Gene symbol	Target gene	Sequence fw and rev (5′–3′)	Accession number
CYP6AR1	Cytochrome P450 6AR1	CAGGGTGCTATACGAGAGGTTG	XM_623359.6
		ACAAGCACCGATCACGTCAG	
CYP4AV1	Cytochrome P450 4AV1	GCGGAAAGAAAAGCCGAGTG	XM_016912202.2
		CACGATACGCTAGTGGCAGT	
CYP4AA1	Cytochrome P450 4aa1-like	AGGCGTGGGTATATCTCGTTA	XM_026441577.1
		GTTTTCGGGCCATTTAGTTGAG	
CYP4G11	Cytochrome P450 4G11	TCGAAGCCGGTCAAAATGGT	XM_006559341.2
		CAGTGGTATCGTGTCCCTCA	
CYP4AZ1	Cytochrome P450 4AZ1	CTTTTTCCAAGCGTACCACGA	XM_006564367.2
		TGGCAAATCGTTGTCCAATGC	
CYP303A1	Cytochrome P450 303A1	TCCTCCAGGTCCAAAATGGTG	XM_026443771.1
		GCCGCCTGTTCTTTTGTCAT	
THF-DH	Cytosolic 10-formyltetrahydrofolate	TGGGTTTTACTGGGTCTACGC	XM_006563788.3
	dehydrogenase	TCCACAAATAGCCGACCAGC	
Arp1	Actin related protein 1	GCCAACACTGTCCTTTCTG	NM_001185146.1
		AGAATTGACCCACCAATCCA	
Enolase	Enolase	GGTGATGAAGGTGGTTTTGC	XM_026444626.1
		GATGCAGCAACATCCATACC	
GAPDH	GAPDH	GATGCACCCATGTTTGTTTG	XM_393605.7
		TTGCAGAAGGTGCATCAAC	
RPL13a	60S ribosomal protein L13a	TGGCCATTTACTTGGTCGTT	XM_623810.5
		GAGCACGGAAATGAAATGGT	
RPS18	40S ribosomal protein S1	GATTCCCGATTGGTTTTTG	XM_625101.6
		CCCAATAATGACGCAAACCT	
FMO5	FMO5 Dimethylaniline monooxygenase	AGGTCTATTTGTCCACCCGC	XM_022797929.1
		GCTGGGCAAAAATCCTGTGAG	
CYP3A56	Cytochrome P450 3A56	TTGCTCGTTTTGGTAGCCCT	XM_022849754.1
		TTTCCGCGTCTGCTACCATT	
SAHD	Succinate dehydrogenase	CAAGGGTGTTACCGCTCTGT	XM_022806549.1
		ACACGAAAAGTACGCCCGTC	
NADH	NADH dehydrogenase	GCGCGATTTGTTAAAGGCGA	XM_022804344.1
		ACGCACAGATGGTATGACCC	
HSP90	Heat shock protein 90	TTTGTAACCGACACGAGCTG	XM_022791765.1
		TGTTGAGCGTGTGAAGAAGC	
18S	18S rRNA	TCAATTAAGGGTGTGGGCCG	XM_022831401.1
		TCACTTCCTGTT CGACAGC	

PCR reactions to quantify the cDNA products were conducted in 96-well plates using a PikoReal Real-Time PCR System (Thermo Scientific, Karlsruhe, Germany). One µl 1:5 diluted cDNA from each of the tested samples was used as a template in 10 µl final reaction volume for qPCR reactions using Biozym Blue S Green qPCR Mix (Biozym Scientific GmbH, Hessisch Oldendorf, Germany) and 4 µM of gene specific primers. The cycling conditions were as follows: initial denaturation at 95 °C for 2 min, followed by 40 cycles of a two-step protocol including 5 s at 95 °C and 20 s at 60 °C. Subsequently, a melt-curve dissociation analysis was performed to confirm quality and specificity of each amplicon.

Normalization of expression data was performed using the three most stably expressed reference genes of the gene set (Table 1): *Arp1, Enolase, GAPDH, RPL13a* and *RPS18* for *A. mellifera* and *SAHD, NADH, HSP90* and *18S* for *V. destructor,* respectively, determined by the geNorm algorithm ^45^. These were *GAPDH*, *RPL13a* and *RPS18* in the case of honey bee and *SAHD*, *NADH* and *18S* in the case of varroa mite. The relative gene expression was obtained by calculating the ddCq values between the control and treatment samples. Significant differences between treatment and control groups were assessed using a Mann–Whitney U test in SPSS v25 (IBM Corp., Armonk, NY, United States) to compare treatment medians with respective controls considering p ≤ 0.05 as the threshold. Log2 fold change relative to the control samples was calculated and compared to the RNA-Seq results in order to confirm the expression results.

## Results

### Honey bee

**Figure 1:**
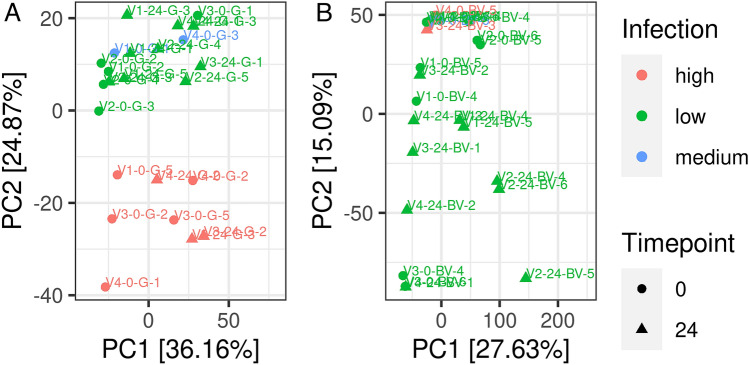
PCA results of (**A**) workers and (**B**) larvae that were highly (red) or moderately (blue) infected or free of infection with Varroa exposed to FA at 0 h (control group, circle) or 24 h (treatment group, triangle).

In total, 48 RNA samples were sequenced from 24 workers and 24 larvae on an Illumina Hi-Seq4000 flow cell. RNA-expression profiles of non-treated (0 h) and treated (24 hpt) samples were assessed. In total, reads could be assigned to 14.380 genes of the honey bee. Few samples showed high infestation by *Varroa*-associated deformed wing virus, indicated by low mapping of sequences to honey bee genome and confirmed by homology search against public databases. Infestation by deformed wing virus led to strong effects on honey bee gene expression profiles as shown by principal component analysis (PCA) of RNA-Seq data (Fig. 1). To avoid a bias in the results on the influence of FA on gene expression, these samples were excluded from further comparative analysis (workers = ten samples; larvae = three samples).

The PCA indicated that most of the variance could be explained by the first two (workers) respectively three (larvae) principal components. However, the PCA did not result in a clear separation of the control and treatment groups (Supplementary Fig. 1S).

**Figure 2:**
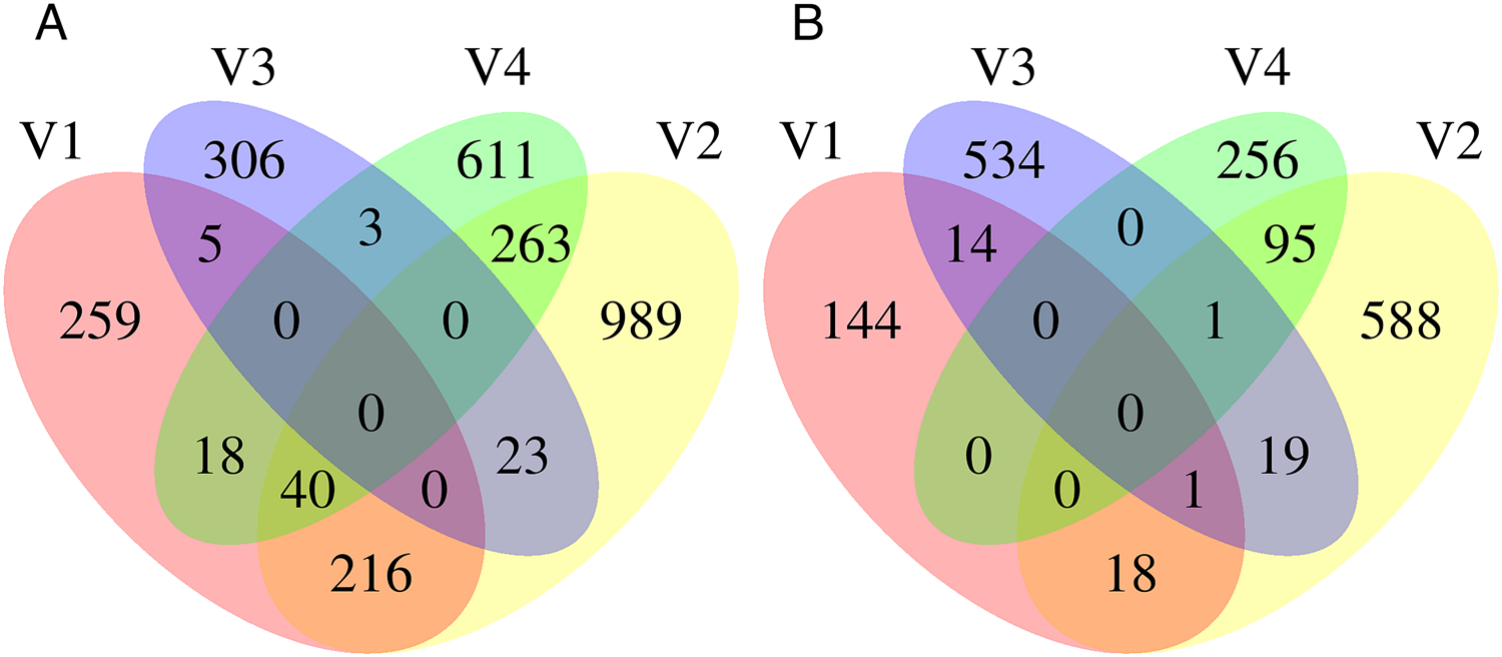
The Venn diagrams show the total number of (**A**) induced and (**B**) repressed genes after FA treatment and the intersection between colonies.

**Figure 3:**
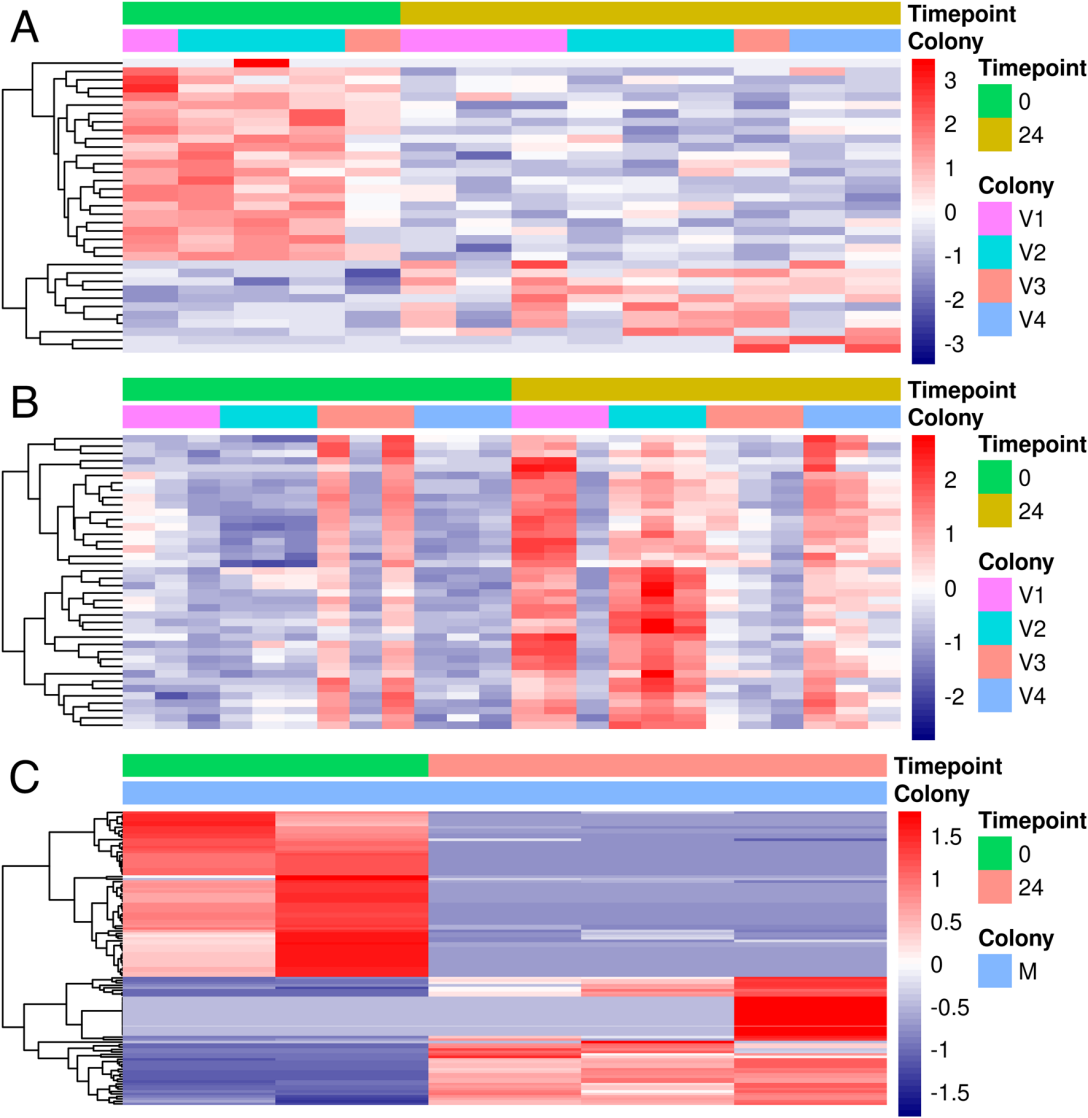
Heatmap summarizing the RNA-seq data of differentially expressed genes between control and treatment group (fold change > 2, p < 0.05) in (**A**) workers, (**B**) larvae and (**C**) Varroa. Red indicates up-regulation and blue indicates down-regulation.

Statistical analysis revealed 35 differentially expressed genes in FA treated workers (24 hpt): 11 induced, while 24 were repressed compared to untreated workers (0 h). Induced genes included detoxification-related CYP450 monooxygenase *CYP6AR1* and *cytosolic 10-formyltetrahydrofolate dehydrogenase* (*FDH*), while repressed genes among others were associated with chitin metabolic processes and chitin binding (*Mucin-3A-like*) and developmental processes (*CYP303A1*) (Fig. 3a). In honey bee larvae, principle component one showed a clear separation of hive number two to other hives. Contributors of this separation include a cluster of four fibroins which suggested different larval age^46,47^ (Supplementary Fig. 3S). For this reason, larval transcriptomes were analysed per hive and genes regulated in multiple hives were intersected. A total of 2733 genes was induced in at least one hive, between 715 and 2253 genes in individual hives respectively (Fig. 2a). Of these, 568 genes were induced in at least two conditions whereas only 40 were induced in larvae of hive one, two and four. No gene was induced across all hives and only 31 out of 337 genes induced in hive three showed induction in other hives (Fig. 2a). On the other hand, 1.670 genes were repressed in larvae of at least one hive. None of which was repressed in larvae more than two hives (Fig. 2b).

Genes induced in three hives included upregulated structural constituents of the cuticle (*Cuticular protein 17, Cuticular protein 28, Cuticle protein 7, Chitotriosidase-1-like, Cell division protein ZipA*). Among the genes with higher variation between colonies one, two and four was *CYP4AA1* a detoxification-associated enzyme (Fig. 3b). A summary of all genes differentially expressed in all pairwise comparisons is shown in Supplementary Tables S1 and S2.

To determine the biological significance, we performed an enrichment analysis of GO annotated differentially expressed genes. Significantly (p ≤ 0.01) enriched GO terms were categorized as "biological processes", "molecular functions" and "cellular components".

**Figure 4:**
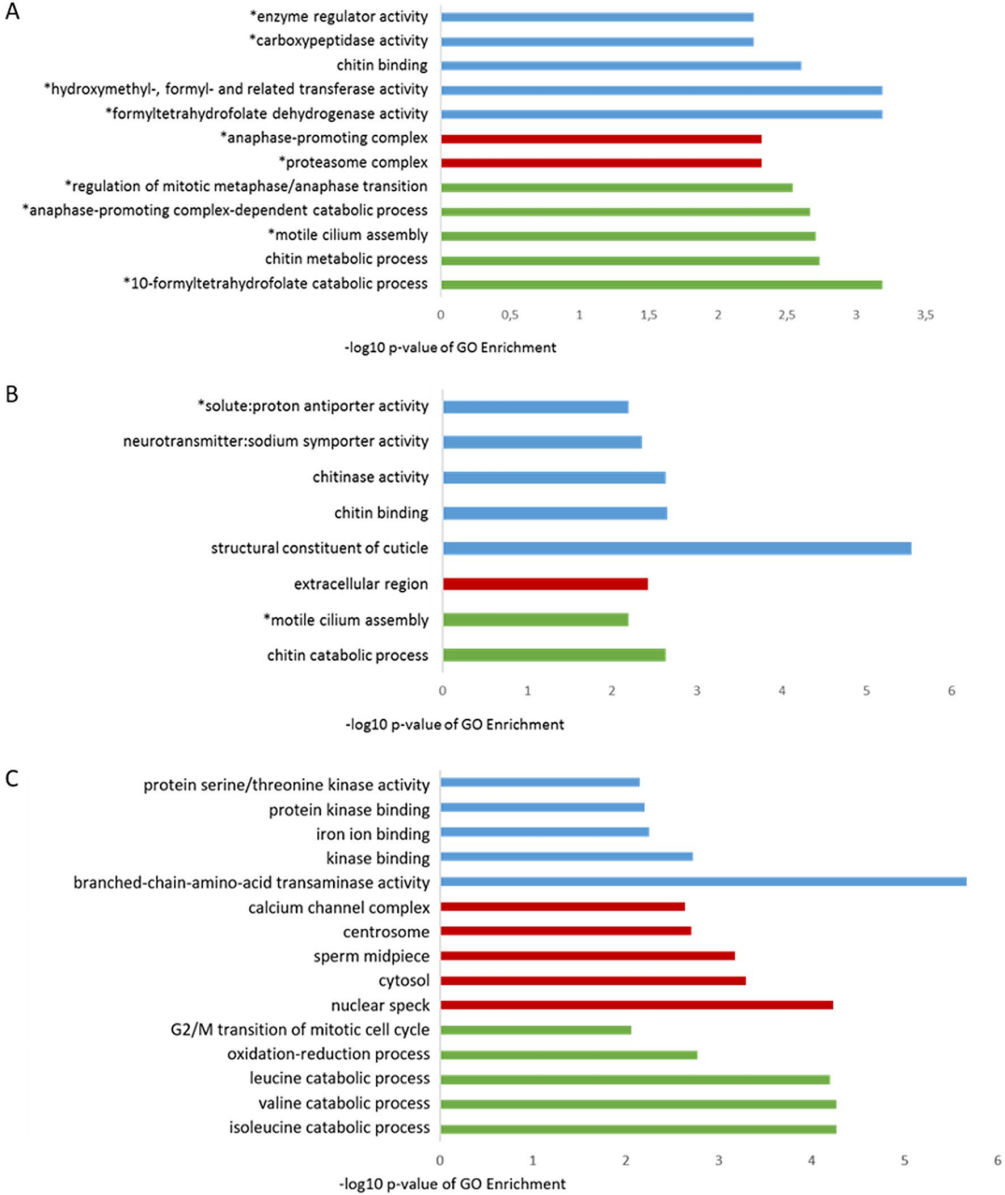
Enriched top five or all significantly overrepresented GO terms of differentially expressed genes of *A. mellifera* (**A**) workers and (**B**) larvae and (**C**) Varroa treated with FA (24 hpt). Molecular functions are shown in blue, cellular components are shown in red and biological processes are shown in green. GO terms marked with stars indicate that only one gene is accumulated in each of these categories.

32% of the differentially expressed genes after FA exposure in the group of workers and 34.21% in the group of larvae could be assigned to GO term annotations (Fig. 4). In workers, FA exposure led to seven over-represented biological processes, eight molecular functions and two cellular components. Among the top enriched GO terms were *10-formyltetrahydrofolate catabolic process* and *chitin metabolic process* in biological processes, the *formyltetrahydrofolate dehydrogenase activity* and *hydroxymethyl-, formyl- and related transferase activity* in molecular functions as well as *proteasome complex* and *anaphase-promoting complex* in cell components. In larvae, FA regulated genes are overrepresented in two biological processes, five molecular functions and one cellular component. Major enriched GO terms included *chitin catabolic process* and *motile cilium assembly* in biological processes *and structural constituent of cuticle and chitin binding* in molecular functions and *extracellular region* in cellular components.

**Figure 5:**
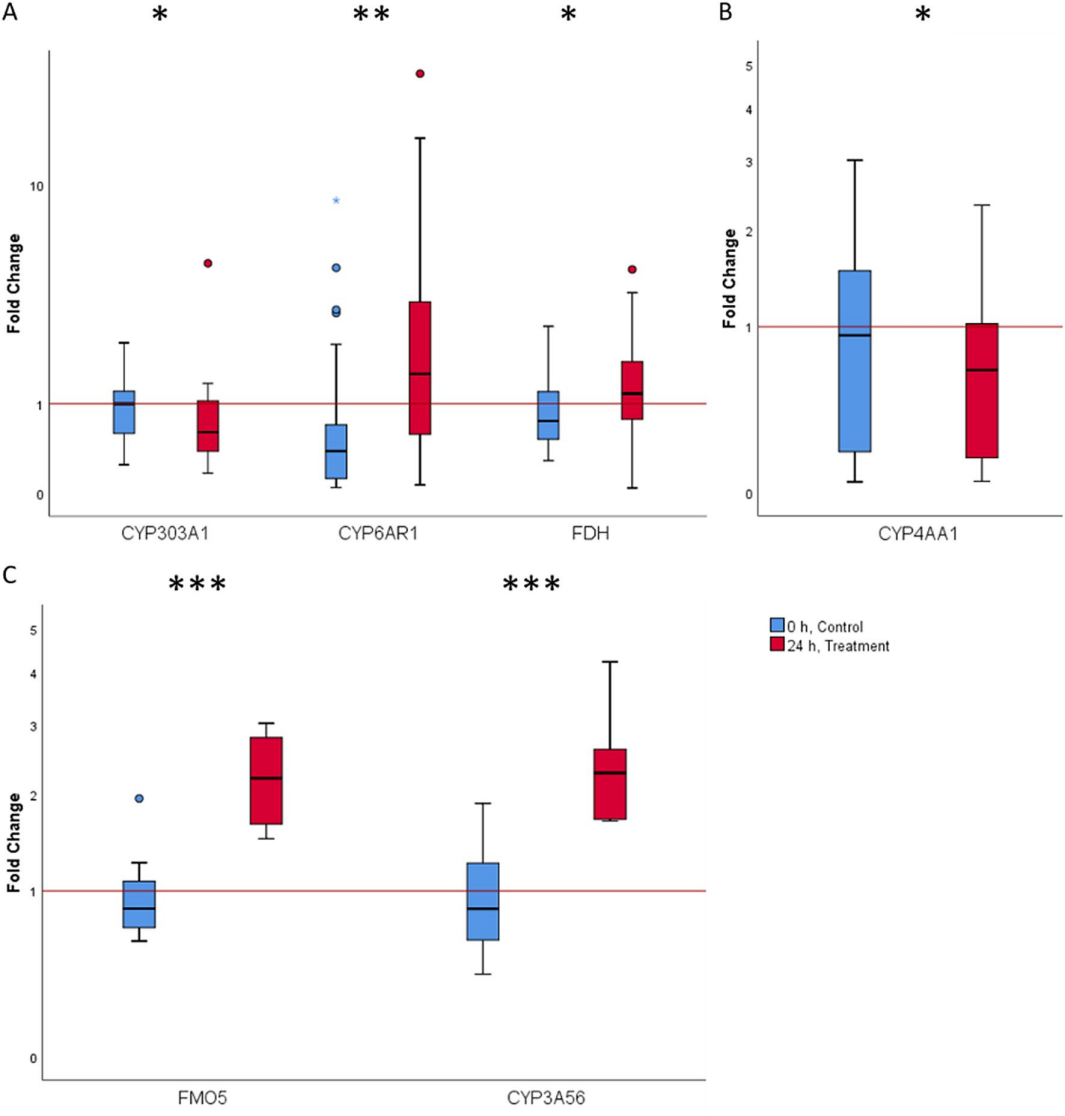
Boxplot analysis of fold changes of the candidate genes from the RT-qPCR analysis for (**A**) workers, (**B**) larvae and (**C**) Varroa that met the criteria (fold change ≥ 2, p-value ≤ 0.05). The line within the box is the median. The upper and lower lines of the box are the first and third quartiles, while the upper and lower whiskers are the 5th and 95th percentile. *p ≤ 0.05; **p ≤ 0.01; ***p ≤ 0.001; Mann–Whitney U-test.

For validation, an RT-qPCR was performed with four candidates that were significantly differentially regulated in RNA-Seq analysis (Fig. 5). Although *CYP4AA1* was not significantly regulated in response to FA exposure, it was selected for further analysis due to its putative interesting biological role. CYP4AA1 belongs to the CYP4 family, which seems to be particularly interesting considering that the number of members is much smaller compared to other insect species (only four genes compared to 32 genes in *D. melanogaster or 34 genes in Nasonia vitripennis*)^48,49^. Similarly, the candidate genes of the honey bee were supplemented with three other genes from the CYP4 family (*CYP4G11, CYP4AV1*, and *CYP4AZ1*). Thus, in the RT-qPCR experiments a total of seven honey bee candidates were investigated. In order to compare the detoxification mechanisms and the different sensitivity of workers and larvae to FA, all candidate genes were equally examined among both age groups. It should be noted that not all of the candidate genes analysed in the RNA-Seq results were differentially regulated in both age groups.

**Figure 6:**
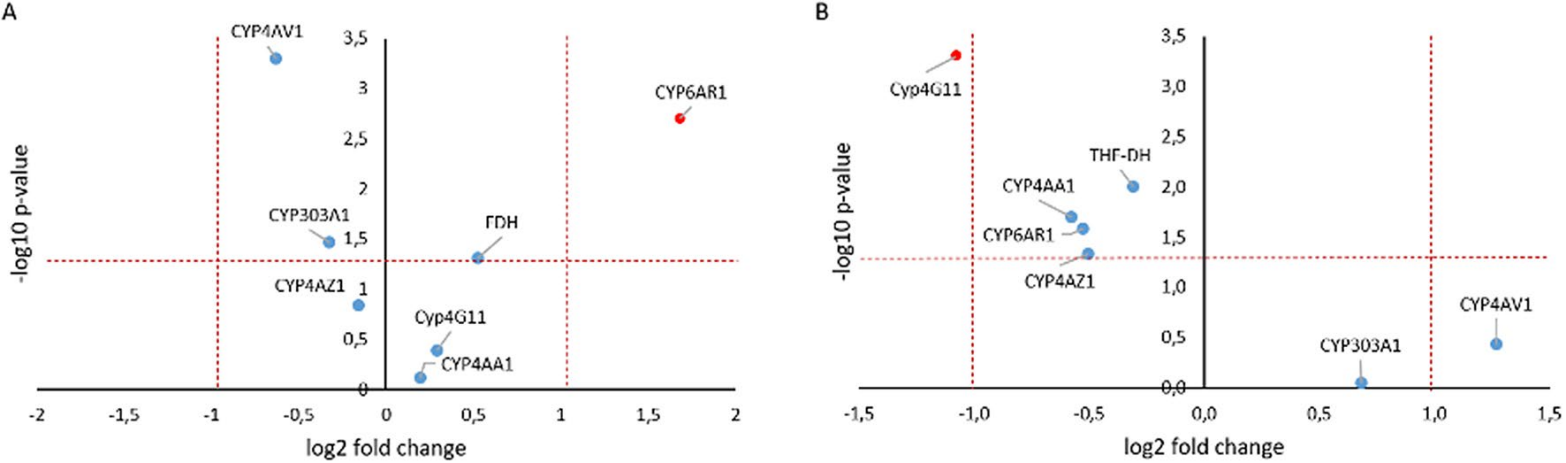
Summary of RT-qPCR data from (**A**) workers and (**B**) larvae of seven differentially expressed transcripts upon FA treatment. A volcano plot analysis of differently expressed genes between the untreated control (0 h) and FA treated group (24 hpt) is plotted on the x-axis (log2 scale), and the statistical significance (p ≤ 0.05) is plotted on the y-axis (− log10 scale). The dotted lines show fold-changes above or below a two-fold up- or down-regulation (values right and left of the vertical lines) and statistical significance (values above the horizontal line).

A significant induction (fold change (FC) ≥ 2, p-value: ≤ 0.05) of the detoxification-associated gene *CYP6AR1* (FC = 3.2, U = 238, Z = − 3.083, p = 0.002) was observed in workers. In the larval group a significant repression of *CYP4G11* (FC = 0.5. U = 170, Z = − 4.698, p < 0.001) was observed 24 hpt (Fig. 5). For some candidate genes, we observed significant regulation but below our initial threshold of a two-fold difference. In the group of workers, these included the repressed gene *CYP4AV1* (by FC = 0.6) and the upregulated *FDH* (by FC = 1.4). In the group of larvae, the repressed gene *FDH* (by FC = 0.8) was included. One gene in the larvae exceeded the FC cut-off without statistical significance (*CYP4AV1*) (Fig. 6).

In some cases, a contradictory regulation could be observed as a reaction to FA treatment. While the workers in most cases experienced a partly significant induction of the detoxification-associated candidate genes, the expression of most candidate genes in the larvae was significantly repressed (Fig. 6).

### Varroa mite

Three pools of each ten non-treated (0 h) and FA treated mites (24 hpt) were sequenced on an Illumina Hi-Seq4000 flow cell. Reads could be assigned to 31.345 transcripts of varroa mites representing 11,852 of 12,868 genes.

FA treatment resulted in 183 gene alterations, 99 genes were induced and 84 repressed. A summary of all genes differentially expressed in all pairwise comparisons is shown in Supplementary Table S3. Remarkably, induced genes were *FMO5 dimethylaniline monooxygenase* and *CYP3A56* as well as the down-regulated genes related to regulation of cellular respiration (*KAPC1-dependent protein kinase catalytic subunit 1*) and oxidative phosphorylation (*ATP synthase subunit mitochondrial, Haloacid dehalogenase-like hydrolase domain-containing protein 2*) (Fig. 3c).

An enrichment analysis of GO-annotated, differently expressed genes revealed that 36.06% of the genes differently expressed after FA exposure could be assigned to GO term annotations (Fig. 4c).

FA-regulated genes were overrepresented in 75 biological processes, 26 molecular functions and 13 cellular components. Among the most frequently enriched GO terms were the *isoleucine* and *valine catabolic process* in biological processes and the *branched-chain amino acid transaminase activity* and *kinase binding* in the molecular functions and the *nuclear spot* and *cytosol in the cellular components*.

The validation by RT-qPCR was performed with two candidate genes that we considered to be potentially biologically relevant.

The results confirmed the significant induction of the detoxification-related genes *FMO5* (FC = 2.25, U = 3, Z = − 3.311, p < 0.001) and *CYP3A56* (FC = 2.38, U = 3, Z = − 3.311, p < 0.001), which were also significantly upregulated in the RT-qPCR analysis (Fig. 5c).

## Discussion

So far, the molecular effects of FA in honey bees have only been investigated in a few studies at gene expression level, e.g. by targeted gene expression analyses^29,30^. For varroa mites, such studies are completely lacking. To our knowledge, this holistic RNA-sequencing study is the first comprehensive and comparative transcriptional analysis that simultaneously examines the effects of FA on *Varroa* and honey bees. Our study addresses the global transcriptome response of total body extracts of honey bees and varroa mites to FA treatment, not the response of specific tissues.

### Honey bee

**Figure 7:**
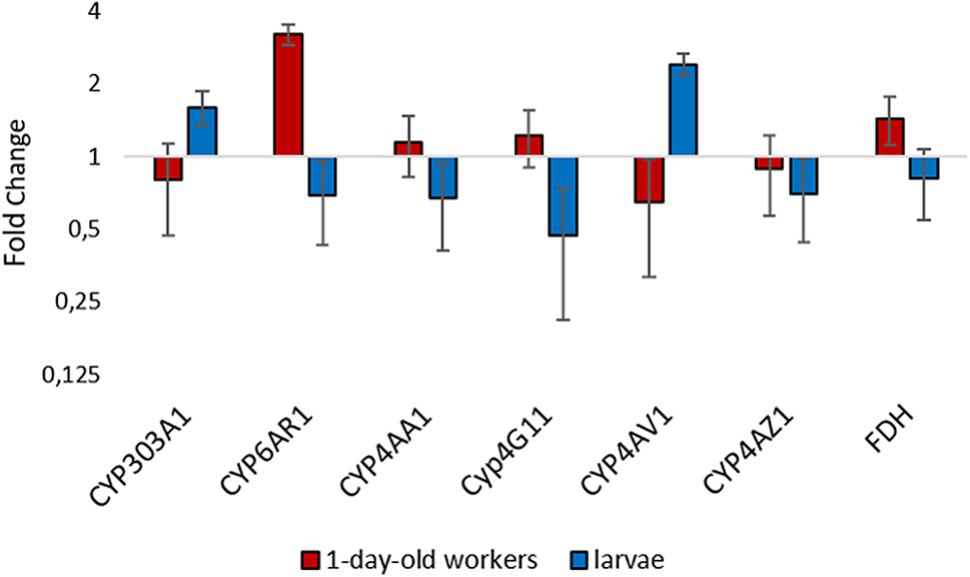
Fold change expression of the candidate genes equally tested between workers (red) and larvae (blue) in the RT-qPCR. Data are mean values ± SEM of the FA treated group (24 hpt). workers n = 60, larvae n = 65.

Comparative studies between the different age groups were carried out to investigate the differential sensitivity to FA treatments that has been described at the phenotypical level in previous reports^16,22,50^. The GO-term enrichment analysis showed differences in transcriptional patterns between the different developmental stages, i.e. worker bees and larvae. Additionally, inverse regulation could be observed for a few genes as a response to the FA treatment. In workers, in most cases an induction of detoxification-associated genes could be observed after FA exposure, whereas in larvae these genes were usually repressed suggesting different sensitivity to FA. One reason for the induction of detoxification enzymes in workers could be their feeding status. Newly emerged workers usually start feeding immediately after hatching, while the capped brood is no longer fed by the workers. It is known that certain honey components (e.g. p-coumaric acid) specifically induce detoxification genes^51^. The feed intake of the workers could therefore have led to an induction of the detoxification enzymes, which could not be observed in the larvae. Furthermore, the detoxification capacity is age-dependent, since with increasing age the metabolic rate and simultaneously the oxidase-specific activity of P450s increases^52,53^. The observed additional inhibition of the expression of detoxification-associated enzymes by FA exposure may increase the harmful effects of other chemical residues and environmental toxins. Combined with the generally lower expression of CYP enzymes in younger bee life stages^54,55^, this may explain the increased sensitivity of bee brood to FA exposure^22,56–58^. Our results may indicate that the younger developmental stages have a reduced ability to induce detoxification-associated enzymes. This could result in a higher sensitivity to FA or to other environmental influences, if the detoxifying enzymes are not FA-specific. (Fig. 7).

The PCA did not allow a clear separation of the treatment and control group by the first principal components, which describe a large part of the overall variance (Supplementary Fig. 2S). From this, we conclude that FA exposure does not have a strong effect on the response of young worker bees. This may be explained by the fact that 60% FA was used for the treatment, which should not harm honey bees when used correctly and under stable environmental conditions as described earlier^59^. There were apparently no incidents during the treatments carried out. No negative effects on honey bee colonies, such as queen or brood loss, were observed. Consequently, the effects of FA on the expression response in bees is considered negligible, so that the separation from untreated individuals with respect to transcription profiles remains rather limited.

Our results show alterations on gene expression levels for different P450 subfamily genes (CYP6 and CYP4) induced by FA treatment. RT-qPCR data confirmed significant up-regulation of *CYP6AR1* (renamed *CYP6AS19*) in workers. Members of the insect-specific CYP6 family of P450s have been associated with insecticide metabolism and resistance in a number of insect pest species^60,61^. Quercetin-metabolizing CYP6AS enzymes (*CYP6AS1, CYP6AS3, CYP6AS4, CYP6AS10*) are known to be induced by constituents of bee products like honey and pollen and in part by the acaricides coumaphos and fluvalinate^62–65^. The gene expression of other members of the CYP6AS family is induced by the varroacide thymol or the pesticide imidaclopri^29,66^. However, it remains to be shown if FA is directly or indirectly involved in the up-regulation of *CYP6AR1*. Furthermore, our results showed an alteration of CYP4 gene expression in workers and larvae. For honey bees only four members of the CYP4 family are reported^48,67^, which could indicate a loss of environmental response^48^. To date, the functions of the individual CYP4 genes are not well characterised: some CYP4s appear to metabolize xenobiotics^61^, others have been associated with hygiene behaviour^68^ and members of the *CYP4G* subfamily were shown to be involved in cuticular hydrocarbon biosynthesis (Qiu et al., 2012). The RT-qPCR results indicated a slight but significant downregulation of the *CYP4AV1* gene in workers. In contrast to these results, *CYP4AV1* was upregulated in the larvae by a fold change greater 2, but without statistical significance. *CYP4AV1* is a paralog of known CYP4 encoding enzymes associated with lipid metabolism^69^. RT-qPCR analysis revealed a significant downregulation of the *CYP4G11* gene in FA exposed larvae. Most insects have two or more CYP4G genes, but there is only one CYP4G gene described in the genome of *Apis* species and in other eusocial bees^49,70,71^. *CYP4G11* is assumed to be an oxidative decarbonylase that metabolizes both long-chain aldehydes for the production of hydrocarbons and short-chain aldehydes that can be used as signalling molecules (Qiu et al., 2012). It is also associated with the clearance of antennas from pheromonal and phytochemical compounds^70^. Another study revealed that *CYP4G11* is probably involved in the chemoperception of environmental chemical signals, as it is strongly expressed in the antennas and legs of foragers but low in both newly emerged workers and nurses^49^. However, other studies assume a significant role of *CYP4G11* in response to oxidative stress and providing protection against oxidative damage^54^. Suppression of P450 enzymes in honey bees challenged by FA exposure has been demonstrated before, and was attributed to a possible negative feedback when the expression of genes of other potential FA detoxification pathways was increased^30^.

In most mammalian species, the major metabolic route of FA is a folate-dependent pathway beginning with the combination of FA with tetrahydrofolic acid (THF) and resulting in the formation of 10-formyltetrahydrofolate (10-THF), catalysed by *10-formyltetrahydrofolate synthetase* (*MTHFD1*). This step is followed by the NADP^+^-dependent conversion of 10-THF into CO_2_ and THF, catalysed by a *cytosolic 10-formyltetrahydrofolate dehydrogenase* (*FDH*)^72–74^. Our RT-qPCR validation confirmed the up-regulation of *FDH* in workers, although RNA-Seq and RT-qPCR results differed in the magnitude of expression. Reasons for a slight difference in the strength of the expression are explained below. In larvae, however, a statistically significant downregulation of this gene was observed. The downregulation of the gene expression of this enzyme could to some extend explain the increased sensitivity of the younger larvae to FA. However, a proteome investigation of honey bees infected with female adult varroa mites showed that the differently expressed proteins in the haemolymph also included the induced FDH, though at a rather low peptide/protein score^75^. Future studies should therefore biochemically investigate whether these transcriptional changes correlate with FA metabolizing enzyme activity in honey bees.

None of the remaining genes tested showed a statistically significant change in the RT-qPCR validation that met our threshold criteria of a > twofold change. However, the activity of detoxification enzymes can be regulated by various mechanisms. On the one hand, by xenobiotic-mediated induction, which is associated with an increase in transcription (and subsequently translation)^76^. This is associated with a measurable change in the cellular mRNA concentration. Nevertheless, this is a relatively slow process, but would result in detectable transcript levels at the chosen point in time after 24 h FA-exposure. On the other hand, activators that enhance the enzyme effect by an activation or stimulation mechanism directly influencing enzyme activity^76^. However, in contrast to induction, they do not influence the cellular concentration of the enzyme and therefore cannot be detected at the mRNA or protein level. This means that a potential detoxification performance by activated enzymes can be significantly higher than we were able to demonstrate in this work.

Although this is the first study to compare the molecular response of honey bees together with the varroa mites in one experiment, other studies have already investigated FA-induced gene expression changes in honey bees only. In previous studies it was found that FA induced gene expression of *PKA-C1*^29^ as well as *AChE* and *Def-1*^30^, while no such alterations were found at significant levels in our study. In addition, FA is believed to lead to suppression of *MRJP-1* and *CYP9Q3* expression^30^. This observation could not be confirmed by our experiments, most probably due to different experimental approaches chosen between studies. Gashout et al.^30^, used older adult bees, while we used freshly hatched workers and larvae. Boncristiani et al.^29^ also carried out field treatments in their study, which, however, differed from our method in the type of FA application. They used Mite Away Quick Strips, i.e. FA polysaccharide gel strips, which remain in the hive for 7 days. The sampling times also differed between the studies: Boncristiani et al.^29^ collected bees from the brood nest before and 30 days after the first treatment, while we analysed the individuals already 24 hpt. In contrast to our study, Gashout, et al.^30^ conducted their experiments under laboratory conditions and applied individual LD05 and LD50 doses topically to the bees, i.e. following a rather artificial laboratory design. Most of the differences between our results and the other studies can probably be explained by the shorter exposure time in our study, the type of application and the exposure of the different FA-sensitive life stages.

### Varroa mite

#### Influence on transcripts associated with detoxification

RT-qPCR data confirmed that *FMO5 dimethylaniline monooxygenase* (*FMO5*) was expressed significantly higher in FA treated mites (24 hpt) than in untreated controls (0 h). Flavin-containing monooxygenases (FMOs) with a FAD prosthetic group represent a family of xenobiotic-metabolizing enzymes^77^. Five mammalian types of FMO are now known as *FMO1*–*FMO5*^77^, all associated with the phase I detoxification of xenobiotic compounds including enzymatic alteration of toxin structure and inhibiting interaction with lipophilic targets in a variety of organisms^67,78^. FMOs can oxidize synthetic therapeutic drugs and herbal alkaloids or other natural products. A number of structurally different compounds containing a “soft nucleophile” (usually nitrogen or sulphur) that have access to the peroxyflavin intermediate are considered as potential substrates^79,80^. The detoxification mechanisms have been associated with the development of resistance to certain chemical pesticides including pyrethroids, pyrrolizidine alkaloids and diamides^81–83^. Both structural and physiological properties of the FMO enzyme family are still relatively unknown, except for functions in xenobiotic metabolism. FMO5 has been detected in the liver of rodents and humans, but according to the literature it cannot easily be classified as a drug-metabolizing enzyme^84^. Nevertheless, the induced expression of this enzyme after FA exposure in our study suggests a role in the detoxification of FA.

RT-qPCR data confirmed differential expression of *CYP3A56* in *Varroa* after FA exposure. In vertebrates, the CYP3A subfamily of the cytochrome P450 superfamily plays a dominant role in metabolic clearance of numerous substances including toxins, pesticides and therapeutic drugs^85,86^. Most studies on CYP3A have been conducted in vertebrates, while little is known about non-vertebrate species, especially varroa mites.

Further studies are required to validate whether altered transcription of these genes is a specific or secondary response to FA.

**Figure 8:**
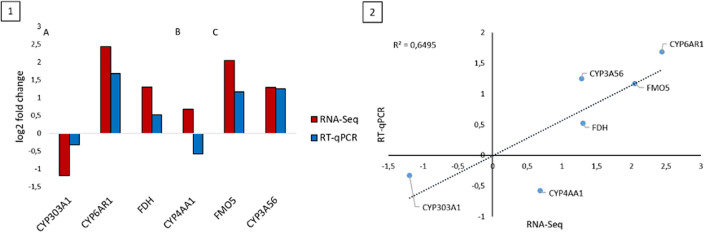
Comparison of RNA-Seq and RT qPCR validation data. (1) Log twofold change. The quantitative measurement of gene expression was determined with RT-qPCR compared to RNA-Seq for seven genes: (**A**) workers, (**B**) larvae and (**C**) Varroa. (2) Correlation analysis between RNA-Seq and RT-qPCR log twofold change. nA = 60, nB = 65, nC = 17.

As a result of validation, the expression changes of most genes were consistent between RNA-Seq and RT-qPCR data. CYP4AA1 was the only target that showed a large difference in expression level between the two methods (Fig. 8). However, already after the cluster analysis of the RNA-Seq results, it was rather variably regulated, which might explain the large difference. However, there are other reasons for minor differences in the results of the two methods: The experiments were conducted in two different years. The test conditions (hive type, colony strength, type of application, etc.) were constant in both years, but other external factors (temperature, humidity, colony specific differences) may have led to slight differences in the effective conditions. To additionally confirm the biological conclusions about the treatments, the validation experiments were supplemented with different biological replicates from the same populatio^87^. Differences between these samples may be another reason for slight differences in the results (Fig. 3). Furthermore, technical differences between the methods such as normalization could explain the variation in the detected magnitude of gene expression profiling^88,89^.

#### Influence on transcripts associated with cellular respiration

After FA exposure, genes that are associated with oxidative phosphorylation and the regulation of cellular respiration according to GO were repressed. This principally confirms previous knowledge from the literature, which describes an inhibition of mitochondrial electron transport by FA binding to cytochrome c oxidase^27^. By inhibiting the respiratory chain, mitochondrial ROS production could be induced, which is known to cause irreversible cell damage and even cell death^90–92^. Although our data do not directly confirm the inhibition of cytochrome c oxidase, they do indicate mitochondrial dysfunction (with repressed regulation of cellular respiration and reduced ATP production), so that the hypothesis of Song and Scharf^93^ could be supported of FA having a neuroexcitatory effect on neurons.

In summary, our data show for the first time endogenous effects of FA treatment on gene expression in both honey bees and varroa mites. Future studies should investigate whether these observed transcriptional changes are also reflected at the protein level and whether the transcriptional pattern and function of candidates identified is FA-specific. If the detoxifying enzymes are indeed FA-specific, this could explain the relatively mild effects of FA treatment on honey bee colonies compared to mites under applied conditions. The influence on further developmental stages of mites and honey bees as well as the molecular response at additional time points are other interesting aspects to be tested in the future. Our results suggest that the molecular basis of the higher FA sensitivity of larvae compared to newly emerged workers is due to limitations in the induction of detoxification capacity. Of particular interest is the specific upregulation of FDH in worker bees, which is known to be involved in mammalian formate metabolism. Our experimental approach showed no overlap of FA-induced detoxification related candidate genes between honey bees and varroa mites, which to a certain extent suggests selectivity differences between these two organisms that could explain the observed differences in toxicity.

## Supplementary Information

Supplementary Information.
