# Supplementary material for: Comparative transcriptomics indicates endogenous differences in detoxification capacity after formic acid treatment between honey bees and varroa mites
Source: Sci Rep. 2020 Dec 14;10:21943. doi: 10.1038/s41598-020-79057-9 (PMC7736338; doi:10.1038/s41598-020-79057-9)

Supplementary

Comparative transcriptomics indicates endogenous differences in detoxification capacity after formic acid treatment between honey bees and varroa mites.

Antonia Genath, Soroush Sharbati, Benjamin Buer, Ralf Nauen, Ralf Einspanier

Table S1: Differentially expressed genes of workers

Induced transcripts

ID	RefSeq Symbol	Description
GB51383	LOC550965	probable Cytochrom P450 6a14
GB40836	LOC100576126	uncharacterized LOC100576126
GB41097	LOC724565	trypsin-7
GB41306	LOC551369	actin, clone 205-like
GB42514		
GB43892	LOC551401	cytosolic 10-formyltetrahydrofolate dehydrogenase
GB46197	LOC726277	anaphase-promoting complex subunit CDC26-like
GB50989		
GB51146	LOC102655756	PDZ and LIM domain protein 7-like
GB51218	LOC107964586	uncharacterized LOC107964586
GB54099		

Repressed transcripts

ID	RefSeq Symbol	Description
GB47752	LOC727290	probable Cytochrom P450 303a1
GB40053	LOC413550	neuropeptide CCHamide-2 receptor-like
GB40123	LOC726599	angiotensin-converting enzyme-like
GB40167	LOC409963	6-phosphofructo-2-kinase/fructose-2,6-bisphosphatase
GB40541	LOC409089	vesicular inhibitory amino acid transporter
GB40610	LOC100578276	uncharacterized LOC100578276
GB42737	LOC724395	mucin-3A-like
GB43585	LOC102656613	uncharacterized LOC102656613
GB44545	LOC107964461	uncharacterized LOC107964461
GB46900	LOC726857	MORN repeat-containing protein 3-like
GB47092	LOC724483	coiled-coil domain-containing protein 65-like
GB47494		
GB48006	LOC724803	membrane metallo-endopeptidase-like 1
GB48383	LOC107965117	uncharacterized LOC107965117
GB48447	LOC100576223	myb-like protein X
GB48938	LOC413574	26S proteasome non-ATPase regulatory subunit 2-like
GB48972	LOC410739	teneurin-m
GB50363	LOC100576540	vacuolar protein sorting-associated protein 27-like
GB51407		
GB51475	LOC102656217	mucin-3A-like
GB51671	LOC411290	uncharacterized LOC411290

GB53073	LOC102654965	uncharacterized LOC102654965
GB53871		
GB54124	LOC100578862	dynein assembly factor 1, axonemal homolog

Table S2: Differentially expressed genes of larvae

Induced transcripts

ID	RefSeq Symbol	Description
GB49626	LOC100577883	Cytochrom P450 4aa1-like
GB40609	LOC102655710	proline-rich extensin-like protein EPR1
GB40842	LOC100576547	glucose dehydrogenase [FAD, quinone]-like
GB40945	LOC552636	dipeptidase 1
GB40997	LOC411021	UDP-glucuronosyltransferase 2B18
GB41188	LOC724865	ABC transporter G family member 20-like
GB42540	LOC100576841	uncharacterized LOC100576841
GB42553	LOC100577098	enolase-phosphatase E1
GB42556	LOC100577380	protein takeout-like
GB42557	LOC100577380	protein takeout-like
GB42582	LOC100579047	cuticular protein 10
GB42594	LOC100578664	organic solute transporter alpha-like protein
GB42598	LOC100578625	tetra-peptide repeat homeobox protein 1-like
GB42792	LOC409805	uncharacterized LOC409805
GB42795	LOC100577064	uncharacterized LOC100577064
GB42800	LOC727028	uncharacterized LOC727028
GB43098	LOC410272	cationic amino acid transporter 2
GB43298	LOC725019	loricrin-like
GB43579	LOC102653655	mucin-19-like
GB43738	PPO	phenoloxidase subunit A3
GB44064	LOC725703	uncharacterized LOC725703
GB44145	LOC409789	uncharacterized peptidase C1-like protein F26E4.3
GB44146	LOC409789	uncharacterized peptidase C1-like protein F26E4.3
GB44560	LOC725148	uncharacterized LOC725148
GB45654	LOC410096	gamma-glutamyltranspeptidase 1-like
GB45973	LOC410638	aromatic-L-amino-acid decarboxylase
GB46277	LOC409277	acidic phospholipase A2 PA4
GB46310	CPR17	cuticular protein 17
GB48123	LOC102654335	uncharacterized LOC102654335
GB48492	JHBP-1	take-out-like carrier protein
GB48981	LOC726451	cuticle protein 7
GB49287	LOC725283	uncharacterized LOC725283
GB49401	LOC725783	protein msta-like
GB49578	LOC552078	glutamyl aminopeptidase-like
GB49616	LOC102656714	chitotriosidase-1-like
GB50206	LOC100578286	flavin-containing monooxygenase FMO GS-OX-like 2

GB50439	LOC725547	cell division protein ZipA
GB50449	LOC100578672	uncharacterized LOC100578672
GB50509	LOC410621	multiple epidermal growth factor-like domains protein 10
GB50526	LOC410626	sodium-coupled monocarboxylate transporter 1
GB51772	LOC102654436	uncharacterized LOC102654436
GB51834	LOC725420	sodium-dependent nutrient amino acid transporter 1-like
GB51845	LOC102654427	sodium-dependent nutrient amino acid transporter 1-like
GB51989	LOC107963967	serine protease inhibitor 3-like
GB52047	LOC102654959	myb-like protein Q
GB52161	CPR28	cuticular protein 28
GB52656	LOC552154	uncharacterized LOC552154
GB52756	LOC408474	apyrase
GB52810	LOC724293	yellow-x1
GB52820	LOC726953	secretin receptor-like
GB52854	LOC413679	cuticular protein analogous to peritrophins 3-E
GB53319	LOC100576504	uncharacterized LOC100576504
GB53925	LOC724993	uncharacterized LOC724993
GB54396	LOC724552	elongation of very long chain fatty acids protein AAEL008004-like
GB54988	LOC102655961	uncharacterized LOC102655961
GB55268	LOC409238	43 kDa receptor-associated protein of the synapse homolog
GB55355	LOC552124	CD151 antigen
GB55613	LOC100576118	uncharacterized LOC100576118
GB55894	LOC725916	uncharacterized LOC725916

Variably regulated transcripts

ID	RefSeq Symbol	Description
GB40157	LOC408421	uncharacterized LOC408421
GB40393	LOC102656154	uncharacterized LOC102656154
GB42639	LOC107963974	uncharacterized LOC107963974
GB44996	Hex110	hexamerin 110
GB45120	LOC410324	villin-like protein quail
GB45188	LOC552689	estradiol 17-beta-dehydrogenase 11
GB45764	LOC413205	tropomyosin-2-like
GB46077	LOC107963964	uncharacterized LOC107963964
GB46629	LOC100577537	uncharacterized LOC100577537
GB47505	LOC725344	histone H2B-like
GB49392	LOC410370	actin-binding Rho-activating protein-like
GB49580	LOC100577614	uncharacterized LOC100577614
GB53209		
GB54124	LOC100578862	dynein assembly factor 1, axonemal homolog
GB54945	LOC107966060	uncharacterized LOC107966060

Table S3: Differentially expressed genes of varroa mite

Induced transcripts

ID	Description
XM_022787822.1	CREB-binding protein OS=Mus musculus OX=10090 GN=Crebbp PE=1 SV=3
XM_022788480.1	Fatty acyl- reductase 1 OS=Xenopus laevis OX=8355 GN=far1 PE=2 SV=1
XM_022788603.1	Extended synaptotagmin-2 OS=Mus musculus OX=10090 GN=Esyt2 PE=1 SV=1
XM_022788618.1	Extended synaptotagmin-2-A OS=Xenopus laevis OX=8355 GN=esyt2-a PE=2 SV=1
XM_022788646.1	Proton-coupled amino acid transporter 1 OS=Homo sapiens OX=9606 GN=SLC36A1 PE=1 SV=1
XM_022788648.1	P protein OS=Homo sapiens OX=9606 GN=OCA2 PE=1 SV=2
XM_022789197.1	CUGBP Elav-like family member 2 OS=Xenopus tropicalis OX=8364 GN=celf2 PE=2 SV=1
XM_022789501.1	RNA exonuclease 1 homolog OS=Mus musculus OX=10090 GN=Rexo1 PE=1 SV=1
XM_022789560.1	Mucolipin-3 OS=Mus musculus OX=10090 GN=Mcoln3 PE=1 SV=1
XM_022789607.1	Proclotting enzyme OS=Tachypleus tridentatus OX=6853 PE=1 SV=1
XM_022789835.1	Echinoderm microtubule-associated 1 OS=Homo sapiens OX=9606 GN=EML1 PE=1 SV=3
XM_022790115.1	Patatin-like phospholipase domain-containing protein 2 OS=Homo sapiens OX=9606 GN=PNPLA2 PE=1 SV=1
XM_022790187.1	Engulfment and cell motility protein 1 OS=Homo sapiens OX=9606 GN=ELMO1 PE=1 SV=2
XM_022790331.1	Transcription factor AP-2-beta OS=Mus musculus OX=10090 GN=Tfap2b PE=1 SV=2
XM_022790418.1	Engulfment and cell motility protein 1 OS=Homo sapiens OX=9606 GN=ELMO1 PE=1 SV=2
XM_022790467.1	MCES_MACF cap guanine-N7 methyltransferase OS=Macaca fascicularis OX=9541 GN=RNMT PE=2 SV=1
XM_022790548.1	Homeobox-containing protein 1 OS=Mus musculus OX=10090 GN=Hmbox1 PE=1 SV=1
XM_022790906.1	---NA---
XM_022790912.1	Alpha-galactosidase A OS=Homo sapiens OX=9606 GN=GLA PE=1 SV=1
XM_022791023.1	Ubiquitin-conjugating enzyme E2 T OS=Homo sapiens OX=9606 GN=UBE2T PE=1 SV=1
XM_022791151.1	Bifunctional purine biosynthesis protein PURH OS=Gallus gallus OX=9031 GN=ATIC PE=1 SV=1
XM_022792703.1	Zinc finger protein 318 OS=Mus musculus OX=10090 GN=Znf318 PE=1 SV=3
XM_022793166.1	---NA---
XM_022793281.1	Puromycin-sensitive aminopeptidase OS=Homo sapiens OX=9606 GN=NPEPPS PE=1 SV=2
XM_022793709.1	Histone H3 OS=Euplotes crassus OX=5936 PE=3 SV=3
XM_022793946.1	Ecdysone 20-monooxygenase OS=Drosophila melanogaster OX=7227 GN=shd PE=1 SV=3
XM_022794088.1	Afadin OS=Mus musculus OX=10090 GN=Afdn PE=1 SV=3

XM_022794111.1	Protein YAE1 OS=Coccidioides immitis (strain RS) OX=246410 GN=YAE1 PE=3 SV=1
XM_022794454.1	La-related protein 6 OS=Mus musculus OX=10090 GN=Larp6 PE=1 SV=1
XM_022794950.1	Gamma-butyrobetaine dioxygenase OS=Pseudomonas (strain AK-1) OX=29440 PE=1 SV=1
XM_022795078.1	GTP-binding protein 2 OS=Homo sapiens OX=9606 GN=GTPBP2 PE=1 SV=1
XM_022795163.1	Leucine-rich repeat transmembrane protein FLRT3 OS=Mus musculus OX=10090 GN=Flrt3 PE=1 SV=1
XM_022795236.1	Plexin-A4 OS=Homo sapiens OX=9606 GN=PLXNA4 PE=1 SV=4
XM_022795361.1	Proclotting enzyme OS=Tachypleus tridentatus OX=6853 PE=1 SV=1
XM_022795726.1	Protein still isoform SIF type 1 OS=Drosophila melanogaster OX=7227 GN=sif PE=2 SV=2
XM_022796425.1	Down syndrome cell adhesion molecule homolog OS=Gallus gallus OX=9031 GN=DSCAM PE=1 SV=3
XM_022796571.1	BTB POZ domain-containing protein KCTD20 OS=Homo sapiens OX=9606 GN=KCTD20 PE=1 SV=1
XM_022796600.1	---NA---
XM_022797398.1	N-terminal Xaa-Pro-Lys N-methyltransferase 1 OS=Danio rerio OX=7955 GN=ntmt1 PE=2 SV=1
XM_022797471.1	Branched-chain-amino-acid cytosolic OS=Ovis aries OX=9940 GN=BCAT1 PE=2 SV=1
XM_022797472.1	Branched-chain-amino-acid cytosolic OS=Ovis aries OX=9940 GN=BCAT1 PE=2 SV=1
XM_022797474.1	Branched-chain-amino-acid cytosolic OS=Ovis aries OX=9940 GN=BCAT1 PE=2 SV=1
XM_022797518.1	V-type proton ATPase 116 kDa subunit a isoform 1 OS=Mus musculus OX=10090 GN=Atp6v0a1 PE=1 SV=3
XM_022797926.1	FMO5_RATDimethylaniline monooxygenase
XM_022797929.1	FMO5_RATDimethylaniline monooxygenase
XM_022798193.1	Acetyl- acetyltransferase mitochondrial OS=Xenopus laevis OX=8355 GN=acat1-b PE=2 SV=1
XM_022798397.1	Nose resistant to fluoxetine protein 6 OS=Caenorhabditis elegans OX=6239 GN=nrf-6 PE=1 SV=3
XM_022798402.1	Nose resistant to fluoxetine protein 6 OS=Caenorhabditis elegans OX=6239 GN=nrf-6 PE=1 SV=3
XM_022798467.1	Homeobox protein six1b OS=Danio rerio OX=7955 GN=six1b PE=1 SV=1
XM_022798710.1	ZC21C_RATZinc finger C2HC domain-containing protein 1C OS=Rattus norvegicus OX=10116 GN=Zc2hc1c PE=2 SV=1
XM_022798844.1	Serine threonine-protein kinase WNK3 OS=Mus musculus OX=10090 GN=Wnk3 PE=1 SV=3
XM_022799066.1	17-beta-hydroxysteroid dehydrogenase type 6 OS=Bos taurus OX=9913 GN=HSD17B6 PE=2 SV=1
XM_022799397.1	S-adenosylmethionine decarboxylase proenzyme OS=Mesocricetus auratus OX=10036 GN=AMD1 PE=2 SV=1
XM_022799576.1	Cytochrom P450 3A56 OS=Fundulus heteroclitus OX=8078 GN=cyp3a56 PE=2 SV=1
XM_022799815.1	Unconventional myosin-VIIa OS=Mus musculus OX=10090 GN=Myo7a PE=1 SV=2
XM_022799996.1	Golgi-associated plant pathogenesis-related protein 1 OS=Mus musculus OX=10090 GN=Glipr2 PE=1 SV=3
XM_022800563.1	Fibulin-1 OS=Gallus gallus OX=9031 GN=FBLN1 PE=2 SV=2

XM_022800630.1	Post-GPI attachment to proteins factor 2-like OS=Drosophila melanogaster OX=7227 GN=CG7990 PE=2 SV=4
XM_022800736.1	TBC1 domain family member 22B OS=Homo sapiens OX=9606 GN=TBC1D22B PE=1 SV=3
XM_022801022.1	---NA---
XM_022802218.1	---NA---
XM_022802315.1	DDB1- and CUL4-associated factor 4 OS=Homo sapiens OX=9606 GN=DCAF4 PE=1 SV=3
XM_022802327.1	Low molecular weight phosphotyrosine protein phosphatase OS=Bos taurus OX=9913 GN=ACP1 PE=1 SV=3
XM_022802364.1	RNA-dependent RNA polymerase 1 OS=Arabidopsis thaliana OX=3702 GN=RDR1 PE=2 SV=1
XM_022802403.1	---NA---
XM_022802911.1	---NA---
XM_022803720.1	Mid1-interacting protein 1-B OS=Danio rerio OX=7955 GN=mid1ip1b PE=2 SV=1
XM_022803739.1	Cyclin-dependent kinase inhibitor 1 OS=Mus musculus OX=10090 GN=Cdkn1a PE=1 SV=4
XM_022804329.1	and PH domain-containing protein 4 OS=Homo sapiens OX=9606 GN=FGD4 PE=1 SV=2
XM_022804398.1	---NA---
XM_022804485.1	Snake venom vascular endothelial growth factor toxin barietin OS=Bitis arietans OX=8692 PE=1 SV=1
XM_022804751.1	Glucose-6-phosphatase 2 OS=Homo sapiens OX=9606 GN=G6PC2 PE=1 SV=1
XM_022805347.1	Iporin OS=Mus musculus OX=10090 GN=Rusc2 PE=1 SV=2
XM_022805572.1	---NA---
XM_022806799.1	---NA---
XM_022806931.1	---NA---
XM_022807030.1	Zinc finger protein 64 isoforms 1 and 2 OS=Homo sapiens OX=9606 GN=ZFP64 PE=1 SV=3
XM_022807033.1	Sodium channel protein para OS=Drosophila melanogaster OX=7227 GN=para PE=2 SV=3
XM_022807064.1	Divergent protein kinase domain 1A OS=Xenopus laevis OX=8355 GN=dipk1a PE=2 SV=1
XM_022807443.1	Isobutyryl- mitochondrial OS=Mus musculus OX=10090 GN=Acad8 PE=1 SV=2
XM_022807521.1	Coronin-B OS=Dictyostelium discoideum OX=44689 GN=corB PE=1 SV=1
XM_022808320.1	Beta-hexosaminidase subunit beta OS=Homo sapiens OX=9606 GN=HEXB PE=1 SV=3
XM_022808326.1	Serine proteinase stubble OS=Drosophila melanogaster OX=7227 GN=Sb PE=2 SV=2
XM_022808514.1	Glutamate-gated chloride channel OS=Drosophila melanogaster OX=7227 GN= Ialpha PE=1 SV=2
XM_022809307.1	Endoribonuclease dcr-1 OS=Caenorhabditis elegans OX=6239 GN=dcr-1 PE=1 SV=3
XM_022810045.1	Sodium hydrogen exchanger 7 OS=Arabidopsis thaliana OX=3702 GN=NHX7 PE=1 SV=1
XM_022810098.1	Zinc transporter 9 OS=Mus musculus OX=10090 GN=Slc30a9 PE=1 SV=2
XM_022810442.1	Protein kibra OS=Drosophila erecta OX=7220 GN=Kibra PE=3 SV=1

XM_022810721.1	Acetylcholine receptor subunit alpha-like 1 OS=Drosophila melanogaster OX=7227 GN=nA alpha1 PE=2 SV=2
XM_022810931.1	Poly -specific ribonuclease PARN OS=Danio rerio OX=7955 GN=parn PE=1 SV=2
XM_022811161.1	Folylpolyglutamate mitochondrial OS=Cricetulus griseus OX=10029 GN=FPGS PE=2 SV=1
XM_022811461.1	GTR1_PIGSolute carrier family facilitated glucose transporter member 1 OS=Sus scrofa OX=9823 GN=SLC2A1 PE=2 SV=2
XM_022811462.1	GTR1_PIGSolute carrier family facilitated glucose transporter member 1 OS=Sus scrofa OX=9823 GN=SLC2A1 PE=2 SV=2
XM_022811513.1	Carboxypeptidase Q OS=Xenopus laevis OX=8355 GN=cpq PE=2 SV=1
XM_022811796.1	Ankyrin-2 OS=Mus musculus OX=10090 GN=Ank2 PE=1 SV=2
XM_022811834.1	Transmembrane protein 47 OS=Mus musculus OX=10090 GN=Tmem47 PE=1 SV=1
XM_022812431.1	Transducin-like enhancer protein 3-B OS=Danio rerio OX=7955 GN=tle3b PE=1 SV=2
XM_022812644.1	Vesicle-associated membrane protein synaptobrevin-binding protein OS=Aplysia californica OX=6500 PE=2 SV=1
XM_022812678.1	Myophilin OS=Echinococcus granulosus OX=6210 PE=2 SV=1
XM_022812843.1	Solute carrier family 41 member 1 OS=Mus musculus OX=10090 GN=Slc41a1 PE=2 SV=1
XM_022812845.1	ADAT1_DROM -specific adenosine deaminase 1 OS=Drosophila melanogaster OX=7227 GN=Adat1 PE=1 SV=1
XM_022813267.1	Protein Mpv17 OS=Danio rerio OX=7955 GN=mpv17 PE=2 SV=1
XM_022813445.1	Lysozyme C OS=Colobus guereza OX=33548 GN=LYZ PE=2 SV=1
XM_022813585.1	Inositol oxygenase OS=Mus musculus OX=10090 GN=Miox PE=1 SV=2
XM_022813927.1	Huntingtin OS=Homo sapiens OX=9606 GN=HTT PE=1 SV=2
XM_022814148.1	NADPH-dependent diflavin oxidoreductase 1 OS=Xenopus laevis OX=8355 GN=ndor1 PE=2 SV=1
XM_022814400.1	Protein jagged-1 OS=Homo sapiens OX=9606 GN=JAG1 PE=1 SV=3
XM_022815357.1	Ribonuclease 3 OS=Homo sapiens OX=9606 GN=DROSHA PE=1 SV=2
XM_022815900.1	PDE1C_RATCalcium calmodulin-dependent 3 -cyclic nucleotide phosphodiesterase 1C OS=Rattus norvegicus OX=10116 GN=Pde1c PE=1 SV=1
XM_022816034.1	---NA---
XM_022816223.1	Zinc transporter ZIP1 OS=Danio rerio OX=7955 GN=slc39a1 PE=2 SV=1
XM_022816236.1	Intraflagellar transport protein 46 homolog OS=Danio rerio OX=7955 GN=ift46 PE=2 SV=2
XM_022816508.1	Sphingomyelin phosphodiesterase OS=Homo sapiens OX=9606 GN=SMPD1 PE=1 SV=5
XM_022816682.1	---NA---
XM_022817063.1	Pleckstrin homology-like domain family B member 2 OS=Mus musculus OX=10090 GN=Phldb2 PE=1 SV=2
XM_022817132.1	TM11L_RATTransmembrane protease serine 11B-like protein OS=Rattus norvegicus OX=10116 GN=Tmprss11bnl PE=2 SV=1
XM_022817207.1	Sialin OS=Homo sapiens OX=9606 GN=SLC17A5 PE=1 SV=2
XM_022817573.1	---NA---
XM_022817699.1	4F2 cell-surface antigen heavy chain OS=Oryctolagus cuniculus OX=9986 GN=SLC3A2 PE=1 SV=2

Repressed transcripts

ID	Description
XM_022788580.1	Vam6 Vps39-like protein OS=Mus musculus OX=10090 GN=Vps39 PE=1 SV=1
XM_022789391.1	Acyl- :lysophosphatidylglycerol acyltransferase 1 OS=Mus musculus OX=10090 GN=Lpgat1 PE=1 SV=1
XM_022789492.1	MFS4B_RATSodium-dependent glucose transporter 1 OS=Rattus norvegicus OX=10116 GN=Mfsd4b PE=1 SV=1
XM_022789737.1	---NA---
XM_022789757.1	BDH_RATD-beta-hydroxybutyrate mitochondrial OS=Rattus norvegicus OX=10116 GN=Bdh1 PE=1 SV=2
XM_022789968.1	EAA2_RATExcitatory amino acid transporter 2 OS=Rattus norvegicus OX=10116 GN=Slc1a2 PE=1 SV=2
XM_022790074.1	KAPC1_DROM -dependent protein kinase catalytic subunit 1 OS=Drosophila melanogaster OX=7227 GN=Pka-C1 PE=1 SV=3
XM_022790077.1	KAPC1_DROM -dependent protein kinase catalytic subunit 1 OS=Drosophila melanogaster OX=7227 GN=Pka-C1 PE=1 SV=3
XM_022790191.1	Patched domain-containing protein 3 OS=Mus musculus OX=10090 GN=Ptchd3 PE=1 SV=1
XM_022790349.1	Hormone-sensitive lipase OS=Mus musculus OX=10090 GN=Lipe PE=1 SV=2
XM_022790435.1	DNA-binding protein P3A2 OS=Strongylocentrotus purpuratus OX=7668 PE=1 SV=1
XM_022790495.1	DNA excision repair protein ERCC-6-like OS=Danio rerio OX=7955 GN=ercc6l PE=1 SV=1
XM_022790528.1	RNA-binding protein Rsf1 OS=Drosophila melanogaster OX=7227 GN=Rsf1 PE=1 SV=1
XM_022790821.1	Purine nucleoside phosphorylase OS=Homo sapiens OX=9606 GN=PNP PE=1 SV=2
XM_022790947.1	NADH dehydrogenase
XM_022790955.1	Actin-binding protein IPP OS=Mus musculus OX=10090 GN=Ipp PE=2 SV=3
XM_022791616.1	E3 ubiquitin-protein ligase RNF19A OS=Mus musculus OX=10090 GN=Rnf19a PE=1 SV=2
XM_022791831.1	Splicing factor 3B subunit 1 OS=Homo sapiens OX=9606 GN=SF3B1 PE=1 SV=3
XM_022791891.1	Helicase ARIP4 OS=Xenopus tropicalis OX=8364 GN=rad54l2 PE=2 SV=1
XM_022792531.1	Thyroid transcription factor 1 OS=Canis lupus familiaris OX=9615 GN=TITF1 PE=2 SV=1
XM_022792747.1	NINJ2_RATNinjurin-2 OS=Rattus norvegicus OX=10116 GN=Ninj2 PE=2 SV=1
XM_022793023.1	---NA---
XM_022793090.1	PDZ and LIM domain protein Zasp OS=Drosophila melanogaster OX=7227 GN=Zasp52 PE=1 SV=2
XM_022793987.1	---NA---
XM_022795612.1	Cell division cycle protein 27 homolog OS=Mus musculus OX=10090 GN=Cdc27 PE=1 SV=1
XM_022796155.1	Thyrotropin receptor OS=Homo sapiens OX=9606 GN=TSHR PE=1 SV=2
XM_022796249.1	---NA---

XM_022796698.1	ATP synthase subunit mitochondrial OS=Caenorhabditis elegans OX=6239 GN= PE=3 SV=1
XM_022796905.1	Required for meiotic nuclear division protein 1 homolog OS=Homo sapiens OX=9606 GN=RMND1 PE=1 SV=2
XM_022797020.1	PPID_RHIO9Peptidyl-prolyl cis-trans isomerase D OS=Rhizopus delemar (strain RA 99-880 ATCC MYA-4621 FGSC 9543 NRRL 43880) OX=246409 GN=cyp12 PE=3 SV=1
XM_022797071.1	phosphoenolpyruvate synthase OS=Bacillus subtilis (strain 168) OX=224308 GN=pps PE=3 SV=1
XM_022797397.1	N-terminal Xaa-Pro-Lys N-methyltransferase 1 OS=Danio rerio OX=7955 GN=ntmt1 PE=2 SV=1
XM_022797522.1	V-type proton ATPase 116 kDa subunit a isoform 1 OS=Mus musculus OX=10090 GN=Atp6v0a1 PE=1 SV=3
XM_022798112.1	Dual serine threonine and tyrosine protein kinase OS=Tetraodon nigroviridis OX=99883 GN=dstyk PE=2 SV=1
XM_022798303.1	N6-adenosine-methyltransferase catalytic subunit OS=Homo sapiens OX=9606 GN=METTL3 PE=1 SV=2
XM_022798823.1	Intersectin-1 OS=Xenopus laevis OX=8355 GN=itsn1 PE=1 SV=2
XM_022798925.1	Protein RCC2 homolog OS=Danio rerio OX=7955 GN=rcc2 PE=2 SV=1
XM_022799441.1	Ribosome biogenesis regulatory protein homolog OS=Mus musculus OX=10090 GN=Rrs1 PE=1 SV=1
XM_022799831.1	UDP-galactose transporter senju OS=Drosophila melanogaster OX=7227 GN=senju PE=1 SV=2
XM_022800174.1	Monocarboxylate transporter 6 OS=Homo sapiens OX=9606 GN=SLC16A5 PE=2 SV=1
XM_022802350.1	NFX1-type zinc finger-containing protein 1 OS=Mus musculus OX=10090 GN=Znfx1 PE=1 SV=3
XM_022802395.1	Protein unc-79 homolog OS=Homo sapiens OX=9606 GN=UNC79 PE=2 SV=4
XM_022802661.1	Probable glutamine--tRNA ligase OS=Drosophila melanogaster OX=7227 GN=Aats-gln PE=2 SV=1
XM_022802685.1	Neurocalcin homolog OS=Drosophila melanogaster OX=7227 GN=Nca PE=1 SV=2
XM_022802687.1	Neurocalcin homolog OS=Drosophila melanogaster OX=7227 GN=Nca PE=1 SV=2
XM_022803071.1	Membrane-associated protein Hem OS=Drosophila melanogaster OX=7227 GN=Hem PE=2 SV=1
XM_022804262.1	Protein ECT2 OS=Mus musculus OX=10090 GN=Ect2 PE=1 SV=2
XM_022804569.1	Ran-binding protein 3 OS=Pongo abelii OX=9601 GN=RANBP3 PE=2 SV=1
XM_022804657.1	High affinity cAMP-specific 3 -cyclic phosphodiesterase 7A OS=Homo sapiens OX=9606 GN=PDE7A PE=1 SV=2
XM_022805204.1	Phosphatidylinositol 4-phosphate 3-kinase C2 domain-containing subunit alpha OS=Pongo abelii OX=9601 GN=PIK3C2A PE=2 SV=1
XM_022805465.1	Rab proteins geranylgeranyltransferase component A OS=Drosophila melanogaster OX=7227 GN=Rep PE=1 SV=1
XM_022805499.1	DNA-directed RNA polymerase III subunit RPC1 OS=Homo sapiens OX=9606 GN=POLR3A PE=1 SV=2
XM_022806623.1	---NA---
XM_022806674.1	Protein capicua homolog OS=Homo sapiens OX=9606 GN=CIC PE=1 SV=2
XM_022806690.1	Chitotriosidase-1 OS=Homo sapiens OX=9606 GN=CHIT1 PE=1 SV=1

XM_022807162.1	Hexosaminidase D OS=Mus musculus OX=10090 GN=Hexd PE=1 SV=1
XM_022807710.1	---NA---
XM_022807949.1	Voltage-dependent L-type calcium channel subunit beta-2 OS=Homo sapiens OX=9606 GN=CACNB2 PE=1 SV=3
XM_022808130.1	Glutaredoxin-related protein mitochondrial OS=Danio rerio OX=7955 GN=glrx5 PE=2 SV=1
XM_022808601.1	5 -AMP-activated protein kinase subunit beta-1 OS=Bos taurus OX=9913 GN=PRKAB1 PE=2 SV=3
XM_022808671.1	Degenerin-like protein unc-105 OS=Caenorhabditis elegans OX=6239 GN=unc-105 PE=1 SV=3
XM_022809849.1	E2AK3_RATEukaryotic translation initiation factor 2-alpha kinase 3 OS=Rattus norvegicus OX=10116 GN=Eif2ak3 PE=1 SV=1
XM_022809894.1	UPF0472 protein C16orf72 homolog OS=Xenopus tropicalis OX=8364 GN= PE=2 SV=1
XM_022810212.1	GTP-binding protein Rheb OS=Homo sapiens OX=9606 GN=RHEB PE=1 SV=1
XM_022810286.1	Ran-binding protein 9 OS=Mus musculus OX=10090 GN=Ranbp9 PE=1 SV=1
XM_022810335.1	Lysosomal-trafficking regulator OS=Mus musculus OX=10090 GN=Lyst PE=1 SV=1
XM_022810464.1	Homeotic protein Sex combs reduced OS=Drosophila melanogaster OX=7227 GN=Scr PE=1 SV=5
XM_022810950.1	Kelch-like protein 36 OS=Bos taurus OX=9913 GN=KLHL36 PE=2 SV=1
XM_022811354.1	Lissencephaly-1 homolog OS=Ixodes scapularis OX=6945 GN= _ISCW007420 PE=3 SV=2
XM_022812181.1	Interleukin-1 receptor-associated kinase 4 OS=Bos taurus OX=9913 GN=IRAK4 PE=2 SV=1
XM_022812248.1	Pleckstrin homology domain-containing family H member 1 OS=Homo sapiens OX=9606 GN=PLEKHH1 PE=2 SV=2
XM_022812337.1	Polyhomeotic-like protein 2 OS=Mus musculus OX=10090 GN=Phc2 PE=1 SV=1
XM_022812471.1	Serine palmitoyltransferase 2 OS=Mus musculus OX=10090 GN=Sptlc2 PE=1 SV=2
XM_022812598.1	Gamma-secretase subunit pen-2 OS=Drosophila melanogaster OX=7227 GN=pen-2 PE=1 SV=3
XM_022812965.1	GTP-binding protein 1 OS=Bos taurus OX=9913 GN=GTPBP1 PE=2 SV=2
XM_022813269.1	Protein Mpv17 OS=Danio rerio OX=7955 GN=mpv17 PE=2 SV=1
XM_022813389.1	Pecanex-like protein 1 OS=Homo sapiens OX=9606 GN=PCNX1 PE=1 SV=2
XM_022813464.1	Zinc finger protein ush OS=Drosophila melanogaster OX=7227 GN=ush PE=1 SV=2
XM_022813719.1	Thymidylate kinase OS=Arabidopsis thaliana OX=3702 GN=ZEU1 PE=2 SV=1
XM_022814033.1	---NA---
XM_022814435.1	Methionine aminopeptidase 1 OS=Danio rerio OX=7955 GN=metap1 PE=2 SV=2
XM_022814473.1	Casein kinase I isoform alpha OS=Xenopus laevis OX=8355 GN=csnk1a1 PE=1 SV=1
XM_022814840.1	Haloacid dehalogenase-like hydrolase domain-containing protein 2 OS=Bos taurus OX=9913 GN=HDHD2 PE=2 SV=1

XM_022814975.1	Zinc finger protein 596 OS=Homo sapiens OX=9606 GN=ZNF596 PE=2 SV=2
XM_022816245.1	---NA---
XM_022816516.1	Zinc finger protein 629 OS=Mus musculus OX=10090 GN=Znf629 PE=2 SV=2
XM_022816864.1	NSFL1 cofactor p47 OS=Pongo abelii OX=9601 GN=NSFL1C PE=2 SV=1
XM_022817068.1	SNW domain-containing protein 1 OS=Pongo abelii OX=9601 GN=SNW1 PE=2 SV=1
XM_022817429.1	Plipastatin synthase subunit D OS=Bacillus subtilis (strain 168) OX=224308 GN=ppsD PE=1 SV=2

Figure 1S: PCA results of honey bees. Gene expression changes were studied in (A) workers and (B) larvae exposed to FA at 0 h (control group, red) or 24 h (treatment group, blue). The results show the relationship between the variables and the gene expression profiles of the different sample time points (untreated control and FA treated group) and colonies.

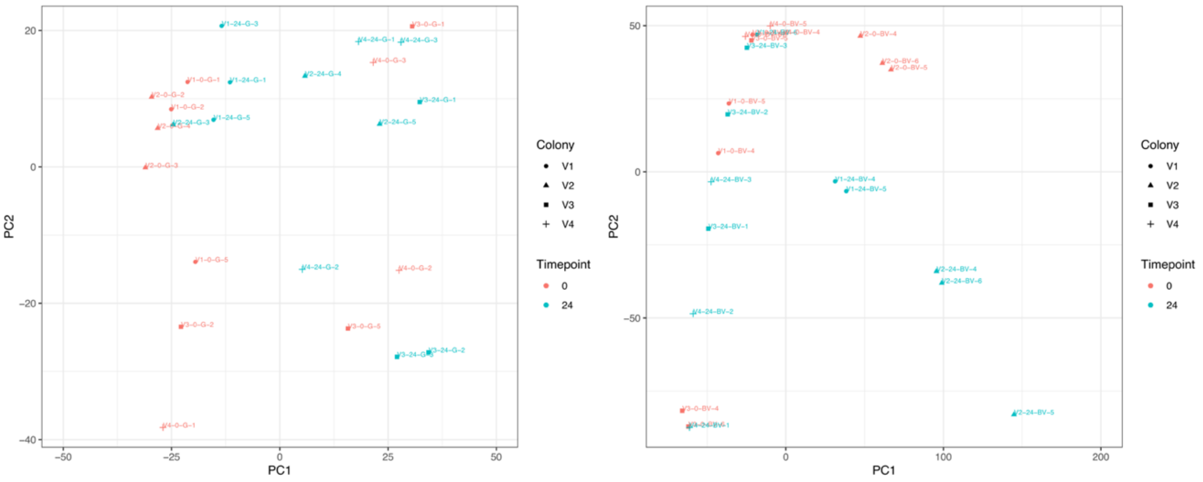

Figure 2S: PCA results of Varroa. The results show the relationship between the variables and the gene expression profiles of the different sample time points (control and treatment group).

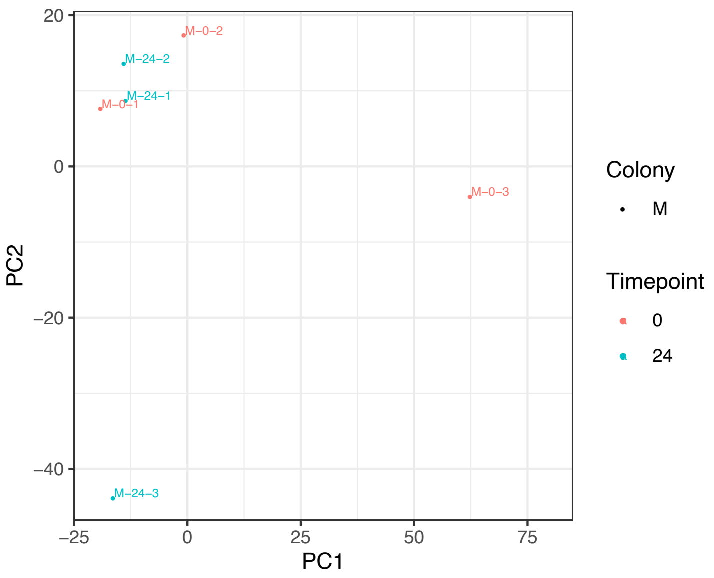

Fig 3S: Heatmap of top and bottom 10 variable loadings of the PCA of larval samples.

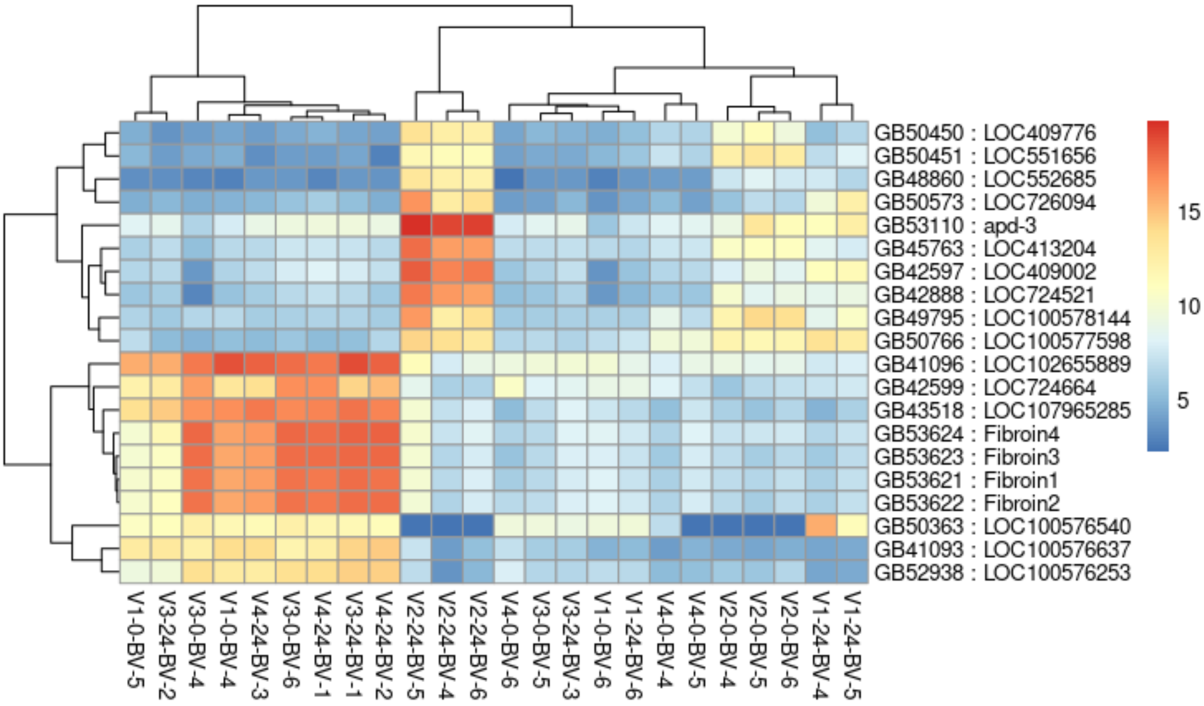

Supplement: Supplementary file 1 — Supplementary Information. [file 41598_2020_79057_MOESM1_ESM.pdf]
